# Use of Irreversible Electroporation in Pancreatic Cancer Patients: A Multi-Center Experience

**DOI:** 10.3390/curroncol32100574

**Published:** 2025-10-16

**Authors:** Bart Hendrikx, Eline-Alice Brys, Alexandra Dili, Thomas Apers, Vera Hartman, Martin Brichard, Filip Gryspeerdt, Claude Bertrand, Geert Roeyen, Frederik Berrevoet

**Affiliations:** 1 Department of General and HPB Surgery and Liver Transplantation, Ghent University Hospital, 9000 Gent, Belgium; bart.hendrikx@uza.be (B.H.); filip.gryspeerdt@uzgent.be (F.G.); 2 Department of Hepatobiliary, Transplant and Endocrine Surgery, Antwerp University Hospital, 2650 Edegem, Belgium; vera.hartman@uza.be (V.H.); geert.roeyen@uza.be (G.R.); 3 Faculty of Medicine and Health Sciences, Ghent University, 9000 Gent, Belgium; elinealice.brys@ugent.be; 4 Department of Digestive, Endocrine and General Surgery, CHU-UCL Namur, Site of Mont Godinne, 5530 Namur, Belgium; alexandra.dili@chuuclnamur.uclouvain.be (A.D.); martin.brichard@chuuclnamur.uclouvain.be (M.B.); cl.bertrand@chuuclnamur.uclouvain.be (C.B.); 5 Department of Surgery, ZAS Hospital, 2030 Antwerp, Belgium

**Keywords:** pancreatic cancer, irreversible electroporation, margin accentuation, tumor ablation, outcomes

## Abstract

**Simple Summary:**

Pancreatic ductal adenocarcinoma has a poor prognosis, and irreversible electroporation (IRE) has emerged as a promising non-thermal ablation technique for locally advanced and borderline resectable pancreatic cancer. This multi-center retrospective study evaluated outcomes in 35 patients treated with IRE for either tumor destruction in unresectable cases or margin accentuation during resection in borderline resectable cases. Our findings highlight the need for careful patient selection and suggest IRE may play a role in managing unresectable disease but not in margin accentuation during surgery. Future research should refine indications for IRE and could inform clinical guidelines, influencing surgical decision-making and improving treatment strategies for pancreatic cancer.

**Abstract:**

Background: Pancreatic ductal adenocarcinoma (PDAC) has a poor prognosis, with a 5-year survival rate of 10%. Irreversible electroporation (IRE), a non-thermal ablative technique, may improve outcomes in locally advanced (LAPC) and borderline resectable pancreatic cancer (BRPC). This multi-center retrospective study aims to evaluate postoperative complications, 90-day mortality, and survival following IRE. Methods: 35 pancreatic cancer patients were treated with IRE between 2015 and 2023 across three Belgian hospitals. IRE was performed for tumor destruction in unresectable LAPC (n = 13) (IRE-LAPC) and for margin accentuation during resection in BRPC (n = 22) (IRE-MA). Primary endpoints were 90-day mortality, complications, and survival (only 33 patients included); secondary endpoints included metastases, local recurrence, and R0-resection rates. Results: Postoperative complications occurred in 23.1% (IRE-LAPC) and 68.2% (IRE-MA) of patients. Overall survival at 24 months was 27.3% (IRE-LAPC) and 27.3% (IRE-MA). Median survival time was 12.7 months (IRE-LAPC) and 13.3 months (IRE-MA). Six patients (17.1%) died within 90 days. Metastasis occurred in 51.5% of patients after a median time of 9.8 months. Local recurrence was seen in 24.2% of patients after a median time of 7.5 months. R0 resection was achieved in 63.6% (IRE-MA). Discussion: IRE for margin accentuation in BRPC is associated with relatively high morbidity and mortality rates and cannot be considered beneficial. In unresectable LAPC, IRE appears relatively safe for local disease control. Further research should clarify patient selection and optimize its therapeutic role.

## 1. Introduction

Pancreatic cancer affects approximately 270,000 patients globally [1] and has become the fourth leading cause of cancer-related death in Western countries [2]. More than ninety percent of PC cases are identified as pancreatic ductal adenocarcinoma (PDAC), with only twenty percent deemed eligible for surgical resection at the time of diagnosis [3,4]. With a 5-year survival rate of around 10%, PDAC is highly aggressive with a poor prognosis [5]. Radical resection remains one of the most critical prognostic factors for survival.

Recently, new surgical devices have been explored in patients with PC to enhance overall survival and improve local disease control. Among these, irreversible electroporation (IRE) has emerged as a promising technique for the treatment of LAPC [6,7,8,9,10] and is currently being assessed for its potential use in borderline resectable pancreatic cancer (BRPC) and resectable pancreatic cancer (RPC) [11,12,13,14,15]. IRE employs high-voltage electric pulses to create nanoscale pores in cell membranes, resulting in disruption of the cell membrane and apoptosis of the pancreatic cancer tissue within the targeted area. Unlike thermal techniques, IRE is a non-ablative treatment that does not cause any thermal damage. Therefore, it can preserve surrounding vital tissues and reduce the risk of vessel or duct injury [16]. IRE has recently been introduced into clinical practice for pancreatic cancer as an adjunct to pancreatectomy, chemotherapy, and radiochemotherapy [6].

In pancreatic cancer surgery, IRE serves dual purposes: it is used both for local tumor destruction and for enhancing resection margins [6]. According to the guidelines [17], tumors with extensive vascular involvement are considered locally advanced and ineligible for surgical resection as complete tumor clearance is not feasible. Alternative treatment options for LAPC include chemoradiation therapy, thermal ablation techniques, or IRE tumor destruction. Treatment for LAPC using IRE has been associated with improved overall survival, with some studies reporting median overall survival of up to 30 months compared to the expected 12 months [18,19,20,21,22]. The recent CROSSFIRE trial aimed to compare the efficacy and safety of MRI-guided stereotactic ablative body radiotherapy versus CT-guided percutaneous irreversible electroporation following standard FOLFIRINOX chemotherapy, but was not able to identify a difference in overall survival or incidence of adverse events between both therapeutic strategies [23]. IRE for LAPC may offer other benefits, such as symptom relief, improved progression-free survival, and potential tumor downsizing [8,16,19,24]. Tumor destruction with IRE can be applied through open laparotomy using ultrasound imaging or percutaneously via CT-guided puncture [25,26]. The open approach may help with probe placement by providing direct visualization of altered anatomy and adhesions.

Additionally, the use of IRE has expanded to margin accentuation for patients with resectable and borderline resectable pancreatic cancer before or after systemic treatment [7,11,12,15]. Unlike other ablative devices, IRE can selectively destroy remaining cancer cells at the surgical margin while preserving surrounding vascular and nervous structures. Achieving an R0 resection is crucial in pancreatic cancer, as the challenge of obtaining clear margins heightens the risk of residual tumor cells in the surrounding tissues [27]. Around 70% of pancreatic cancer surgeries result in R1 resections, which are associated with shorter median survival rates compared to R0 resections [28]. By accentuating surgical margins, IRE could potentially increase the likelihood of successful R0 resections, thereby improving progression-free and overall survival [6,7,20,29]. However, because IRE has multiple immune-mediated effects on the tumor microenvironment [30], an immediate postoperative increase in the R0 resection rate is unlikely. The therapeutic effect of IRE on margin status is delayed, and its use is not expected to increase the proportion of patients achieving R0 resections in the immediate postoperative period. Although IRE has shown a positive effect on overall survival, its impact on margin positivity rates, complication rates, and local recurrence is still a matter of debate among various studies [8,11,12,14].

Most studies on the use of IRE for BRPC and LAPC so far have originated from single institutions. This has sparked interest to see whether other centers can reproduce similar outcomes [9,10,20,31]. We conducted a nationwide multi-center analysis to evaluate the use of IRE for both local tumor destruction and margin accentuation across three high-volume hospitals for pancreatic surgery in Belgium. The main objectives were to assess 90-day mortality, postoperative complications, and overall survival associated with both IRE applications.

## 2. Materials and Methods

### 2.1. Patient Selection

A multi-institutional retrospective analysis was performed, evaluating patients with pancreatic cancer who underwent IRE between January 2015 and September 2023. At the time of data collection, the IRE procedures, which included both local tumor destruction and margin accentuation, were performed at three centers in Belgium: Antwerp University Hospital, Mont Godinne Hospital Namur, and Ghent University Hospital. Tumor staging was primarily based on abdominal and chest CT. In cases of diagnostic uncertainty, PET-CT was used, and MRI was selectively employed for more precise staging, mainly to exclude liver metastases.

All patients except for RPC received neoadjuvant chemotherapy with or without radiotherapy, according to institutional protocols. Chemotherapy consisted of either Folfirinox or gemcitabine combined with nab-paclitaxel. After 6–8 cycles of Folfirinox, patients were restaged using CA 19-9 levels and radiological imaging. If CA 19-9 levels remained stable and no radiological evidence of disease progression was observed, surgical exploration was considered and discussed in a multidisciplinary oncological meeting, with consent from the treating oncologist and from the patient. During exploration, patients underwent either margin accentuation with IRE when resection was feasible (IRE-MA group) or local tumor ablation with IRE when resection was not possible (IRE-LAPC group).

According to NCCN criteria [32], LAPC was defined as tumors with ≥180° encasement of superior mesenteric artery (SMA), celiac or hepatic artery; or unreconstructible portal (PV) or superior mesenteric vein (SMV) involvement that precludes a margin-negative resection. BRPC was defined as tumors ≥ 180° involvement of SMV or PV, with proximal and distal vessels not suitable for reconstruction; or <180° encasement of SMA, celiac or hepatic artery. RPC was defined as tumors with no arterial contact and no venous involvement of the SMV and PV, or ≤180° contact without vein contour irregularity.

Patients with BRPC and eligible LAPC who showed a favorable response to neo-adjuvant therapy (e.g., tumor marker reduction, completion of therapy) underwent surgical exploration. When resection was feasible, IRE-MA was performed; otherwise, IRE ablation (IRE-LAPC) was carried out. IRE-LAPC was also offered to LAPC patients intolerant to neoadjuvant therapy or with unreconstructable vascular involvement, as well as to RPC and BRPC patients who declined or were deemed unfit for resection.

Exclusion criteria included the presence of metal implants such as bile duct stents or cardiac pacemakers, and a history of cardiac arrhythmias. More specifically, metallic stents were not considered a contraindication for patients undergoing resection with IRE-MA. However, for patients with LAPC scheduled for local treatment with IRE-LAPC, the presence of metallic stents was a contraindication. Regarding cardiac arrhythmias, only those occurring at the initiation of IRE therapy, as confirmed by electrocardiography (ECG), were deemed contraindications.

At the initiation of the study protocol, a standardized indication for the use of IRE in pancreatic lesions had not yet been reported. Therefore, the inclusion criteria varied across the three university hospitals, with some centers including multiple histological diagnoses and the presence of oligometastatic disease.

The primary endpoint of this study was to assess 90-day mortality, postoperative complications, and overall survival in patients treated with both IRE applications. Secondary endpoints included metastasis-free survival, local progression-free survival, and R0-resection rates.

### 2.2. Data Collection

Medical records and relevant radiological imaging for all patients were thoroughly reviewed. Data on patient demographics, tumor characteristics, survival status, margin positivity, tumor recurrence, and metastasis were retrospectively collected. Margin positivity was defined as an R2 resection or as an indirect or direct R1, meaning the presence of tumor cells within 1 mm or directly to the resected margin [33]. Morbidity and mortality were systematically evaluated through electronic medical records. Morbidity and physical status were defined using the American Society of Anesthesiologists classification (ASA). Postoperative complications were graded using the Clavien–Dindo classification and the comprehensive complication index (CCI). Local tumor recurrence, development of metastasis, and overall survival were defined as the time from the date of the IRE procedure to the first radiological confirmation of local tumor progression, distant metastasis, or death, respectively.

### 2.3. IRE Procedure

Patients included in the IRE-LAPC group underwent local tumor destruction using IRE, and patients in the IRE-MA group underwent IRE margin accentuation. The IRE procedure was conducted using the NanoKnife^®^ system (AngioDynamics, Latham, New York, NY, USA) during laparotomy under general anesthesia. In all three hospitals, surgical procedures were performed by experienced HPB surgeons proficient in intraoperative ultrasound assessment of the liver and pancreas. All initial cases were supported by trained AngioDynamics specialists, who provided on-site technical assistance during the procedures.

Needle placement followed the technique described by Martin et al. [10], using a caudal-to-cranial approach to allow continuous ultrasound guidance and minimize dissection. Probes were placed before excessive dissection of pancreatic head and neck. Probe placement was based on detailed intraoperative measurements of tumor size and surrounding structures, typically involving two to five electrodes positioned 1.5–2.5 cm apart, adjusted for tumor location, size, and proximity to vasculature. Particular care was taken to avoid vascular injury to the SMV, portal vein, SMA, and hepatic artery. Depending on the local tumor situation, different margins were treated, mainly SMV, SMA and posterior margins.

After an initial test phase, treatment pulses were delivered synchronously with the electrocardiogram. IRE needle placement was continuously guided by intra-operative ultrasound. For IRE-LAPC, the probes were positioned around the tumor without resection; for IRE-MA, probes were placed adjacent to planned resection margin(s), followed by resection and reconstruction. Each electrode had an effective field range of up to 2 cm. Although earlier studies have used shorter pulse duration, our protocol applied pulses at 1500 V/m with a pulse wavelength of 100 ms, as previously described [10].

### 2.4. Follow-Up

After the IRE procedure, patients were admitted overnight to either the intensive care unit or the medium care unit. Postoperative complications were documented and graded using the Clavien–Dindo classification system and the comprehensive complication index (CCI). Patients underwent an immediate CT scan post-IRE and were then followed up by routine CT scans of thorax and abdomen every three months during the first year. Patients were scheduled for outpatient clinic visits every three months according to a standardized follow-up protocol, with earlier visits arranged if needed and for surviving patients, the follow-up period extended to 24 months.

### 2.5. Statistical Analysis

Categorical variables were reported as counts (*n*) and percentages (%). Continuous variables with a normal distribution were presented as means and standard deviations (SD), and continuous non-parametric variables were described using medians and interquartile ranges (IQR). The Shapiro–Wilk test was employed to assess normality. Kaplan–Meier curves illustrated overall survival and metastasis-free survival for the IRE-MA and IRE-LAPC groups. All patients were included in the survival analysis except for two cases: one patient with distal cholangiocarcinoma and one patient with pancreatic metastasis from previously resected renal cell carcinoma. These exclusions were made to reduce heterogeneity and to allow survival outcomes to be analyzed specifically for patients with PDAC. All statistical analyses were conducted using SPSS Statistics 29 (IBM corporation, Armonk, NY, USA).

### 2.6. Ethical Approval

This study was approved by the Medical Ethics Committee of Ghent University Hospital (protocol code: BC-10695, date of approval 6 October 2021). All patients gave their informed consent.

## 3. Results

Between January 2015 and September 2023, 35 patients were treated with IRE. Of these, 13 patients (37.1%) were included in the IRE-LAPC group and underwent local tumor destruction, and 22 patients (62.9%) were included in the IRE-MA group and underwent margin accentuation with surgical resection.

### 3.1. Patient Demographics and Preoperative Characteristics

The mean age across the cohort was 65.2 years (SD 8.4), with a predominance of male patients (65.7%, n = 23) (Table 1). Regarding preoperative status, 16 patients (45.7%) were classified as ASA II and 15 (42.9%) as ASA III. The most common comorbidities were diabetes without end-organ damage (n = 11, 31.4%) and peptic ulcers (n = 5, 14.3%), while no comorbidities were present in 16 patients (45.7%). Histopathological analysis showed that 91.4% of the tumors (n = 32) were diagnosed as PDAC. The remaining cases included PDAC arising from intraductal papillary mucinous neoplasm (IPMN) (n= 1, 2.9%), cholangiocarcinoma (n = 1, 2.9%), and pancreatic metastasis of previously resected renal carcinoma (n = 1, 2.9%), the latter initially suspected to be a primary pancreatic adenocarcinoma.

Tumors were primarily located in the head and body of the pancreas in 19 (57.5%) and 11 patients (33.3%), respectively. The mean tumor size was 31.9 mm (SD 11.1). The superior mesenteric artery (SMA) was involved in 20 patients (57.1%), and the portal vein and/or superior mesenteric vein (SMV) in 17 patients (48.6%). In the IRE-MA group, the SMA was identified as the margin to be treated in 63.6% (n = 14) of the cases. 84.6% (n = 11) in the IRE-LAPC group and 86.4% (n = 19) in the IRE-MA group received neoadjuvant treatment. After pre-treatment, most patients (n = 24, 68.6%) in the cohort were staged as LAPC. Nine patients (25.7%) had BRPC, with eight of them (36.4%) treated in the IRE-MA group. 2 patients had primarily resectable disease.

### 3.2. Operative Characteristics

In the IRE-LAPC group, none of the patients underwent tumor resection following IRE tumor destruction (n = 13, 100%) (Table 2). Conversely, all patients in the IRE-MA group (n = 22, 100%) had their tumors surgically resected following IRE margin accentuation. Among these, pancreaticoduodenectomy was the most common procedure, performed in 13 patients (59.1%), followed by total pancreatectomy in four patients (18.2%), and distal pancreatectomy in five (22.7%). Portal vein reconstruction was required in five cases (22.7%). The number of probes varied between groups: a median of five probes was used in the IRE-LAPC group, and a median of three in the IRE-MA group. In the IRE-MA group, R0 resection was achieved in 14 patients (63.6%), and a R1 resection occurred in eight patients (36.4%). No R2 resections were reported.

### 3.3. Postoperative Complications, Follow-Up and Survival Outcomes

The overall complication rate was 51.4%, with a lower rate in the IRE-LAPC group (23.1%, n = 3) compared to the IRE-MA group (68.2%, n = 15). Common complications included gastroparesis (25.7%, n = 9), chyle leakage (22.9%, n = 8), infection (20%, n = 7), and diarrhea (17.1%, n = 6). Pancreatic fistula grade B was seen in two patients (5.7%) and grade C in one (2.9%). One patient (2.9%) experienced GI-bleeding. The median CCI over the cohort was 22.6 (IQR 8.7–40.5), with the IRE-LAPC group showing a median CCI of 8.7 (IQR 0–28.7) and the IRE-MA group a median CCI of 32.7 (IQR 14.6–40.9). Twelve patients (34.3%) experienced a Clavien–Dindo grade III complication or higher. The mean hospital stay was 14.8 days (SD 6.2), with 17.7 days (SD 5.5) in the IRE-MA group and 10.1 days (SD 4.2) in the IRE-LAPC group. Six patients (17.1%) died in the 90-day postoperative period, with four of them in the IRE-MA group (18.2%) and two of them in the IRE-LAPC group (15.4%). Causes of death varied and included NSTEMI, multi-organ failure, hepatic artery thrombosis, cholangitis, aortic dissection, and one unknown etiology. Rehospitalization occurred in 32.4% of patients (n = 11) within three months post-IRE treatment, predominantly in the IRE-MA group (42.9%, n = 9). The primary reasons for readmission were malnutrition (22.9%, n = 8) and diarrhea (14.3%, n = 5). 80% of patients (n = 28) received adjuvant therapy postoperatively. All patients in the IRE-LAPC cohort (n = 13, 100%) received systemic treatment, whereas only 68.2% of patients (n = 15) in the IRE-MA group did. The mean time to initiation of adjuvant therapy for the whole cohort was 47.1 days (SD 15.5), with a mean of 43.4 days (SD 12.6) for the IRE-LAPC group and 52.7 days (SD 15.4) for the IRE-MA group (Table 3).

**Figure 1:**
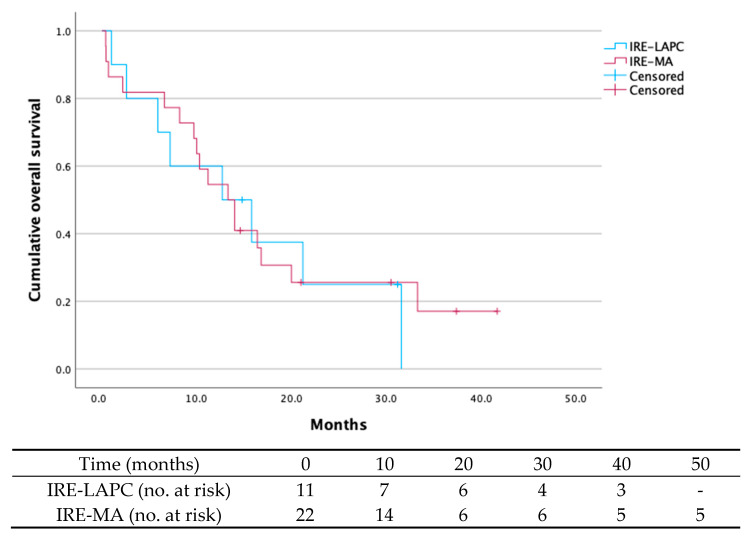
Overall survival of the IRE-LAPC group (n = 11) and the IRE-MA group (n = 22). The overall survival rate for the whole cohort was 27.3% after a 24-month follow-up period. In the IRE-LAPC group and IRE-MA group, overall survival was 27.3% and 27.3%, respectively. The median survival time across the cohort was 13.3 months (95% CI 9.3–17.1), with median survival times of 12.7 months (95% CI 0.8–24.5) for the IRE-LAPC group and 13.3 months (95% CI 9.1–17.5) for the IRE-MA group.

At two-year follow-up (Table 4), 69.7% of patients (n = 23) had died: n = 7 (63.6%) in the IRE-LAPC group and 72.7% (n = 16) in the IRE-MA group. The median survival time across the cohort was 13.3 months (95% CI 9.3–17.1), with median survival times of 12.7 months (95% CI 0.8–24.5) for the IRE-LAPC group and 13.3 months (95% CI 9.1–17.5) for the IRE-MA group (Figure 1).

Local tumor recurrence occurred in 24.2% of patients (n = 8) after a median time of 7.5 months (95% CI 6.0–11.6). In the IRE-LAPC group, 27.3% (n = 3) experienced recurrence after a median time of 9.0 months (95% CI 3.0–9.0). In the IRE-MA group, local recurrence occurred in 22.7% (n = 5) of patients after a median time of 6.0 months (95% CI 6.0–12.0).

**Figure 2:**
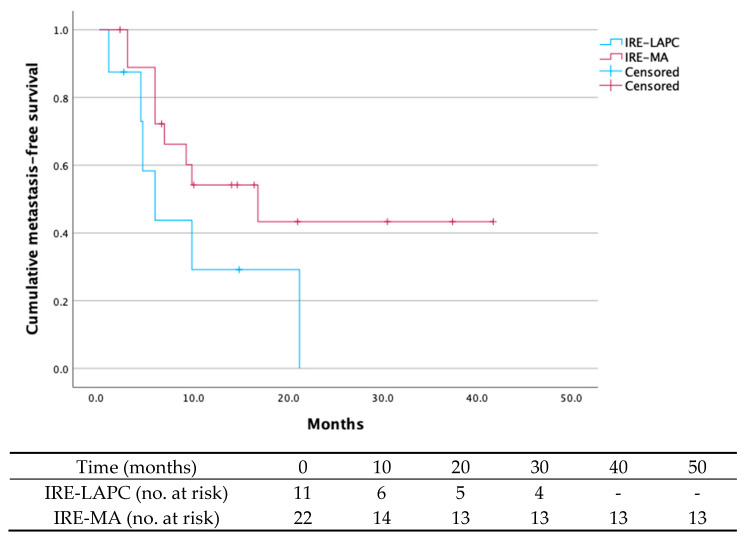
Metastasis-free survival of the IRE-LAPC group (n = 11) and the IRE-MA group (n = 22). The metastasis-free survival rate for the whole cohort was 48.5% after a 24-month follow-up period. In the IRE-LAPC group and IRE-MA group, metastasis-free survival was 45.5% and 59.1%, respectively. The median time until metastasis across cohort was 9.8 months (95% CI 0.6–19.0), with median metastasis times of 5.9 months (95% CI 2.6–9.2) for the IRE-LAPC group and 16.8 months (95% CI 4.7–28.9) for the IRE-MA group.

Across the cohort, metastasis occurred in 51.5% after a median time of 9.8 months (95% CI 1.9–17.7). Among the eleven patients who underwent local tumor destruction, 54.5% (n = 6) developed metastases after a median time of 5.9 months (95% CI 2.6–9.2). In the IRE-MA group, 40.9% (n = 9) of patients developed metastases after a median time of 16.8 months (95% CI 4.7–28.9) (Figure 2).

## 4. Discussion

This is the first nationwide multi-center study in Belgium to explore two distinct applications of IRE in pancreatic cancer treatment, although the study population was limited.

The results of this analysis indicate concerns regarding the safety versus the benefits of margin accentuation with IRE. The complication rate in our IRE-MA cohort was 68.2% (15/22), a considerable incidence that is nonetheless consistent with the previously reported rate of 63% [15]. When examining the severity of these complications using the Clavien–Dindo classification system, 40.9% of patients (9/22) were graded III or higher, a higher percentage than the 19% major complications reported by Ratnayake et al. [8]. Common complications in our cohort were chyle leakage in 36.4%, gastroparesis in 31.8%, infections in 27.3%, and diarrhea in 27.3%. The high occurrence of gastroparesis and diarrhea could possibly be attributed to the IRE procedure by disrupting the nervous plexus around the superior mesenteric artery margin. Further research needs to be carried out to determine whether this is a consistent finding in larger cohorts. As four patients of the IRE-MA group died in the immediate postoperative period (18.2%), this might have also significantly increased the number of Clavien–Dindo grade >III complications.

Suraju et al. [15] reported a significantly higher rate of splanchnic thrombosis in patients undergoing IRE-mediated margin accentuation compared to those undergoing resection alone (22% vs. 5%, *p* = 0.05). They attributed this severe complication to the inflammatory and vascular responses triggered by the electroporation procedure, which caused endothelial injury, subsequently leading to thrombus formation in the affected areas. In our cohort, there were two 90-day postoperative deaths potentially attributable to this inflammatory vascular response triggered by IRE: one due to hepatic artery thrombosis, and another due to aortic dissection. These specific complications have not yet been widely reported in the current literature. The commonly held assumption that tissue integrity is fully preserved by IRE has already been challenged by Brys et al. [34] as well as by multiple animal studies demonstrating injury to surrounding tissues [35,36].

Two other 90-day postoperative death occurred in a patient with significant preoperative comorbidities, making it difficult to definitively attribute the outcome to IRE. These findings suggest that further research is needed to clarify the safety of IRE margin accentuation, particularly for its inflammatory response on the vascular system and for patients with resectable pancreatic cancer who may not be ideal candidates for extensive surgery [37].

Contrary to IRE margin accentuation for patients with borderline resectable pancreatic cancer, local tumor ablation using IRE may represent a generally safe treatment modality for patients with unresectable pancreatic cancer. Postoperative complications occurred in 23.1% of patients in our IRE-LAPC cohort, a result comparable to the 25% rate of adverse events reported by Stephens et al. [19]. Importantly, in their study comparing 201 patients who received IRE tumor destruction with 200 patients who underwent pancreatectomy, the authors suggested that IRE might result in a significantly lower incidence of adverse events compared to pancreatectomy in early-stage disease (25% vs. 39%). However, certain complications seem to be specific to IRE tumor destruction, including infections (25%), ascites (16%), and gastrointestinal bleeding (6%). In our IRE-LAPC cohort, we observed quite low incidences of these complications, with infections observed in 7.7% of cases, ascites in 16%, and no instances of GI bleeding. These discrepancies could be attributed to our smaller sample size, which reduces overall exposure to adverse events. Nonetheless, our findings support the conclusion that IRE for local tumor destruction in unresectable pancreatic cancer can be considered safe for clinical use.

R0 resection is a critical factor in pancreatic cancer treatment, significantly impacting overall survival. Although some studies report improved R0 resection rates with IRE [8,11], others have found no significant effect [12,14]. In our cohort, the R0 rate following IRE margin accentuation was 63.7%, which is comparable to the 73% R0 rate reported by Ratnayake et al. [8] in their systematic review of nine studies involving 235 pancreatic tumors treated with MA-IRE. Our findings suggest that IRE may enhance the likelihood of achieving R0 resections in pancreatic surgery, an essential outcome for improving survival in resectable pancreatic cancer. However, since IRE suppresses tumor growth by enhancing the infiltration of effector CD8+ T-cells [30], an immediate improvement in the R0 resection rate may not be observed post-surgery. Additionally, the cellular apoptosis induced by IRE may not be readily detectable at this stage, making local progression-free survival a more reliable measure of its impact.

In one of the pioneering studies on IRE, Martin et al. [9] demonstrated a significantly longer local progression-free survival with IRE local tumor destruction compared to standard therapy (14 vs. 6 months). Our findings fall in between, showing a median local progression-free survival of 9.0 months in patients who underwent IRE-LAPC, which does not allow for a definitive conclusion on this matter. For margin accentuation with IRE, a recent study from Martin et al. [12] reported an 18% local recurrence rate in their pancreatectomy-alone group compared to 17% in their IRE-MA group. Our IRE-MA cohort yielded similar results, showing a local recurrence rate of 22.7%. The median time to local recurrence in our IRE-MA cohort was 6.0 months, shorter than the 10.5 months observed in the pancreatectomy-alone group in Martin et al. [12]. Although this difference may reflect the smaller sample size, margin accentuation with IRE may not have the potential to prolong time to local recurrence in borderline resectable and resectable pancreatic cancer.

Few studies have focused on metastasis-free survival in patients undergoing IRE, as most focus on local progression and overall survival. However, recent research suggests that IRE may offer long-term benefits through immune cell activation [14], making metastasis-free survival an exciting area for future investigation. In our cohort, 40.9% of patients who underwent margin accentuation developed metastases during follow-up, which is lower than the 65% reported by Martin et al. [12]. Notably, their study, like others, did not report the median time to metastasis. Considering the potential for significant findings in larger prospective studies and the recent hypothesis regarding long-term immune activation by IRE, the median metastasis-free survival in the margin accentuation cohort was 16.8 months (95% CI, 4.7–28.9).

One of the key questions surrounding IRE is its impact on the overall survival (OS). Literature on IRE-MA presents conflicting results regarding OS. Studies by Ratnayake and Martin et al. report median OS rates of 22 and 34.2 months, respectively [8,12,14]. However, other studies, including ours, suggest a less significant survival benefit. Our study found a median OS from procedure of 13.3 months, which may be due to the higher rate of immediate postoperative mortality (18.2%) compared to Ratnayake et al. (8%) [8] and standard pancreatectomy (3.2%) [38]. It is however important to note the possible introduction of time bias since different definitions for median overall survival have been used across studies on IRE. These findings suggest that, while IRE margin accentuation may improve R0 resection rates, its role in improving overall survival remains unclear.

While several studies have reported median OS ranging from 27 to 28 months for IRE-LAPC [25,26,39], other studies provide more conservative estimates of 15 to 17 months [9,40,41,42,43]. In our cohort, the median OS after IRE-LAPC procedure was 12.7 months, which aligns more closely with this data. The one-year mortality rate post-IRE in patients with LAPC remains high, as seen in other studies [18]. It is crucial to refine patient selection criteria to identify those who may benefit most from IRE’s potential survival benefits while minimizing immediate postoperative mortality risks [39].

Several limitations were identified in this retrospective study. First, there is no standardized indication for IRE in pancreatic cancer treatment yet, which resulted in variations in patient inclusion criteria across the three institutions. Specifically, some centers included patients with borderline resectable tumors, while others also treated locally advanced or metastases from other primary cancers. Additionally, criteria regarding tumor size, vascular involvement, or neo-adjuvant treatments (such as chemotherapy or radiotherapy) differed between institutions. These variations could have affected outcomes by introducing heterogeneity in tumor biology and patient fitness, potentially influencing complication rates, local control, and overall survival.

In addition, the power of this study is limited by the small patient populations for both tumor destruction and margin accentuation. Most of the 90-day mortality was attributable to “classical” post-pancreatic surgery complications, including pancreatic fistula, hemorrhage, and multi-organ failure. Given the limited sample size and the heterogeneity of the study population, no analysis could be performed to determine whether IRE influenced these outcomes, nor its effect on the secondary outcomes of this study.

Due to the retrospective nature of this study, our analysis is susceptible to information bias. In addition, the absence of a standardized protocol for patient inclusion introduces a clear risk of selection bias. Furthermore, as the researchers were not blinded, the study may be subject to publication bias and confirmation bias.

## 5. Conclusions

This national retrospective analysis indicates that IRE for margin accentuation in borderline and resectable tumors cannot be considered beneficial due to the high rates of complications and 90-day postoperative mortality. In contrast, IRE for unresectable LAPC appears to be a relatively safe option for local disease control, with a postoperative complication rate of 23.1%. Further research is needed to refine patient selection criteria and to determine whether IRE offers meaningful benefit in pancreatic cancer.

## Figures and Tables

**Table 1:** Patient demographics and preoperative characteristics.

Characteristics	Total n = 35	IRE-LAPC n = 13	IRE-MA n = 22
Age, years [mean (SD)]	65.2 (8.4)	67.9 (9.6)	63.6 (7.4)
Gender			
Men	23 (65.7)	9 (69.2)	14 (63.6)
Women	12 (34.3)	4 (30.8)	8 (36.4)
BMI, kg/m^2^ [(median (IQR)]	24.1 (21.3–26.6)	25.2 (23.3–31.4)	22.5 (21.3–26.4)
ASA-classification			
Class I	3 (8.6)	1 (7.7)	2 (9.1)
Class II	16 (45.7)	4 (30.8)	12 (64.5)
Class III	15 (42.9)	7 (53.8)	8 (36.4)
Comorbidities			
None	16 (45.7)	7 (53.8)	9 (40.9)
Myocardial infarction	2 (5.7)	2 (15.4)	0
Peripheral vascular disease	3 (8.6)	2 (15.4)	1 (4.5)
Cerebrovascular disease	2 (5.7)	1 (7.7)	1 (4.5)
Congestive heart failure	1 (2.9)	1 (7.7)	0
Chronic pulmonary disease	2 (5.7)	0	2 (9.1)
Peptic ulcer	5 (14.3)	0	5 (22.7)
Diabetes without damage to end-organs	11 (31.4)	5 (38.5)	6 (27.3)
Diabetes with damage to end-organs	0	0	0
Tumor without metastasis	4 (11.4)	0	4 (18.2)
Gastric bypass	1 (2.9)	1 (7.7)	0
Histological diagnosis			
PDAC	32 (91.4)	11 (84.6)	21 (95.5)
PDAC following IPMN	1 (2.9)	0	1 (4.5)
Cholangiocarcinoma	1 (2.9)	1 (7.7)	0
Metastasis of other primary	1 (2.9)	1 (7.7)	0
Location tumor			
Head	19 (57.5)	5 (41.7)	14 (66.7)
Body	11 (33.3)	6 (50)	5 (23.8)
Neck	3 (9.1)	1 (8.3)	2 (9.5)
Tail	0	0	0
Tumor size, mm [mean (SD)]	31.9 (11.1)	32.7 (13.3)	29.5 (8.5)
cT stage			
T2	6 (17.1)	1 (7.7)	5 (22.2)
T3	1 (2.9)	0	1 (4.5)
T4	25 (71.4)	11 (84.6)	14 (63.6)
cN stage			
N0	19 (54.3)	4 (30.8)	15 (68.2)
Nn	13 (37.1)	8 (61.5)	5 (22.7)
cM stage			
M0	30 (85.7)	11 (84.6)	19 (86.4)
Mn	2 (5.7)	0	2 (9.1)
Vascular involvement			
None	3 (8.6)	3 (23.1)	0
Portal vein and/or SMV	17 (48.6)	7 (53.9)	10 (45.5)
SMA	20 (57.1)	6 (46.2)	14 (63.6)
Coeliac trunk	10 (28.6)	4 (30.8)	6 (27.3)
Hepatic artery	9 (25.7)	4 (30.8)	5 (22.7)
Resectability			
RPC	2 (5.7)	2 (15.4)	0
BRPC	9 (25.7)	1 (7.7)	8 (36.4)
LAPC	24 (68.6)	10 (76.9)	14 (63.6)
Neoadjuvant treatment regimen	30 (85.7)	11 (84.6)	19 (86.4)
Chemotherapy	30 (85.7)	11 (84.6)	19 (86.4)
Folfirinox	25 (71.4)	10 (76.9)	15 (68.2)
Gemcitabine	5 (14.3)	1 (7.7)	4 (18.2)
Radiotherapy	4 (11.4)	0	4 (18.2)

Data are expressed as n(%) unless otherwise specified. ASA = American Society of Anesthesiologists, Physical Status Classification; PDAC = Pancreatic Ductal Adenocarcinoma; IPMN = Intraductal Papillary Mucinous Neoplasm; SMV = Superior Mesenteric Vein; SMA = Superior Mesenteric Artery; RPC = Resectable Pancreatic Cancer; BRPC = Borderline Resectable Pancreatic Cancer; LAPC = Locally Advanced Pancreatic Cancer.

**Table 2:** Operative characteristics.

Characteristics	Total n = 35	IRE-LAPC n = 13	IRE-MA n = 22
Tumor resected?			
Yes	22 (62.9)	0	22 (100)
No	13 (37.1)	13 (100)	0
Type of surgery			
Pancreaticoduodenectomy	13 (37.1)	-	13 (59.1)
Total pancreatectomy	4 (11.4)	-	4 (18.2)
Distal pancreatectomy	5 (14.3)	-	5 (22.7)
Additional interventions			
Portal vein reconstruction	5 (14.7)	-	5 (23.8)
IRE procedure time, min [mean (SD)]	367 (17.9)	277 (22.8)	420 (17.3)
Number of probes	4 (3–5)	5 (4–6)	3 (2–4)
Electrode spacing, mm [mean (SD)]	15 (0)	15 (0)	15 (0)
pT stage			
T1	5 (14.3)	-	5 (22.7)
T2	8 (24.2)	-	8 (36.4)
T3	4 (11.4)	-	4 (18.2)
T4	4 (11.4)	-	4 (18.2)
Missing	1 (2.9)	-	1 (4.5)
pN stage			
N0	9 (25.7)	-	9 (40.9)
Nn	12 (34.3)	-	12 (64.5)
Missing	1 (2.9)	-	1 (4.5)
pM stage			
M0	7 (20)	-	7 (31.8)
Mn	5 (14.3)	-	5 (22.7)
Missing	10 (28.6)	-	10 (45.4)
Resection margin			
R0 resection	14 (40)	-	14 (63.6)
R1 resection	8 (24.2)	-	8 (36.4)
R2 resection	0	-	0

Data are expressed as n(%) unless otherwise specified.

**Table 3:** Postoperative complications and follow-up.

Characteristics	No. Patients n = 35	IRE-LAPC n = 13	IRE-MA n = 22
Complications	18 (51.4)	3 (23.1)	15 (68.2)
Gastroparesis	9 (25.7)	2 (15.4)	7 (31.8)
Infection	7 (20)	1 (7.7)	5 (27.3)
Liver abscess	1 (2.9)	0	1 (4.6)
Abscess at ablated zone	1 (2.9)	1 (7.7)	0
Cholangitis	3 (8.6)	1 (7.7)	2 (9.1)
Peritonitis	1 (2.9)	0	1 (4.6)
Cellulitis	1 (2.9)	0	1 (4.6)
Other: non-specified	3 (8.6)	0	3 (13.6)
Ascites	2 (5.7)	1 (7.7)	1 (4.6)
Pancreatic fistula	3 (8.6)	2 (15.4)	1 (4.6)
Grade B	2 (5.7)	1 (7.7)	1 (4.6)
Grade C	1 (2.9)	1 (7.7)	0
Colon perforation	2 (5.7)	0	2 (9.1)
Chyle leakage	8 (22.9)	0	8 (36.4)
Portal vein thrombosis	2 (5.7)	1 (7.7)	1 (4.6)
Hepatic artery thrombosis	1 (2.9)	0	1 (4.6)
Diarrhea	6 (17.1)	0	6 (27.3)
Cardiac arrhythmias	3 (8.6)	0	3 (13.6)
GI bleeding	1 (2.9)	0	1 (4.6)
CCI [median (IQR)]	22.6 (8.7–40.5)	8.7 (0–28.7)	32.7 (14.6–40.9)
Severity of complications, Clavien–Dindo grade III or higher	12 (34.3)	3 (23.1)	9 (40.9)
In hospital stay, days [mean (SD)]	14.8 (6.2)	10.1 (4.2)	17.7 (5.5)
Death within 90 days post IRE	6 (17.1)	2 (15.4)	4 (18.2)
Cause of death within 30 days post IRE			
Death of unknown cause	1 (2.9)	0	1 (4.6)
NSTEMI	1 (2.9)	0	1 (4.6)
Hepatic artery thrombosis	1 (2.9)	0	1 (4.6)
Cholangitis	1 (2.9)	1 (7.7)	0
Multi-organ failure	1 (2.9)	1 (7.7)	0
Aortic dissection	1 (2.9)	0	1 (4.6)
Rehospitalization within 3 months post IRE	11 (32.4)	2 (15.4)	9 (42.9)
Reason for rehospitalization			
Malnutrition	8 (22.9)	0	8 (36.6)
Diarrhea	5 (14.3)	0	5 (27.3)
Cholangitis	1 (2.9)	1 (7.7)	0
Sepsis	1 (2.9)	0	1 (4.6)
Pain	1 (2.9)	1 (7.7)	0
Received adjuvant treatment	28 (80)	13 (100)	15 (68.2)
Adjuvant treatment regimen			
Chemotherapy	20 (57.1)	7 (53.9)	13 (59.1)
Radiotherapy	6 (17.1)	5 (41.7)	1 (4.6)
Chemoradiotherapy	2 (5.7)	1 (7.7)	1 (4.6)
Time to adjuvant treatment, days [mean (SD)]	47.1 (15.5)	43.4 (12.6)	52.7 (15.4)

Data are expressed as n(%) unless otherwise specified. CCI = Comprehensive Complication Index; NSTEMI = non-ST elevated myocardial infarct.

**Table 4:** Survival outcomes at 24-month follow-up for all patients except for one patient with distal cholangiocarcinoma and one patient with pancreatic metastasis from previously resected renal cell carcinoma (n = 33).

Characteristics	No. Patients n = 33	IRE-LAPC n = 11	IRE-MA n = 22
Local tumor recurrence at 24 months	8 (24.2)	3 (27.3)	5 (22.7)
Metastases at 24 months	17 (51.5)	6 (54.5)	9 (40.9)
Location of metastases			
Lung	7 (21.2)	3 (27.3)	4 (18.2)
Liver	7 (21.2)	1 (9.1)	6 (18.2)
Peritoneum	8 (24.2)	4 (36.4)	4 (18.2)
Overall survival at 24 months			
Alive	9 (27.3)	3 (27.3)	6 (27.3)
Death	23 (69.7)	7 (63.6)	16 (72.7)
Lost to follow-up	1 (3.0)	1 (9.1)	0
Cause of death			
Tumor progression	10 (30.3)	5 (45.5)	5 (22.7)
Multi-organ failure	7 (21.2)	1 (9.1)	6 (27.3)
Cardial	2 (6.1)	1 (9.1)	1 (4.5)
Cerebrovascular disease	1 (3.0)	0	1 (4.5)
Cholangitis	1 (3.0)	1 (9.1)	0
Euthanasia	1 (3.0)	0	1 (4.5)
Unknown	1 (3.0)	0	1 (4.5)

Data are expressed as n(%) unless otherwise specified.

## Data Availability

The data that support the findings of this study are available at the corresponding author, upon reasonable request.

## Abbreviations

The following abbreviations are used in this manuscript:

IRE	Irreversible Electroporation
PDAC	Pancreatic ductal adenocarcinoma
LAPC	Locally advanced pancreatic cancer
BRPC	Borderline resectable pancreatic cancer
RPC	Resectable pancreatic cancer
IRE-LAPC	Local tumor destruction with IRE in unresectable LAPC
IRE-MA	Margin accentuation with IRE during resection in BRPC and RPC
SMA	Superior mesenteric artery
PV	Portal vein
SMV	Superior mesenteric vein
IPMN	Intraductal papillary mucinous neoplasm
ASA	American Society of Anesthesiologists, Physical Status Classification
CCI	Comprehensive Complication Index
NSTEMI	Non-ST elevated myocardial infarct
